# Clathrin Couture: Fashioning Distinctive Membrane Coats at the Cell Surface

**DOI:** 10.1371/journal.pbio.1000192

**Published:** 2009-09-08

**Authors:** Linton M. Traub

**Affiliations:** Department of Cell Biology and Physiology, University of Pittsburgh School of Medicine, Pittsburgh, Pennsylvania, United States of America

Eukaryotic cells utilize intricate mechanisms for the uptake and intracellular sorting of various macromolecules, such as membrane components and extracellular proteins. Microscopic imaging studies have helped considerably to describe and define important steps of the uptake process. It has been shown that the cytosolic face of the plasma membrane is studded with small, domed assemblages of peripheral membrane proteins. These constitute transitory sorting stations that dynamically remodel the composition of the cell surface in response to both intracellular and extracellular stimuli. Typically, within a minute of forming, these assemblages invaginate to generate “buds” that then detach, generating small (∼60 nm), membrane-bound transport vesicles that deliver their contents (often receptors) to specific intracellular compartments—namely acceptor early endosomes—for further dissemination within the cell (Figure 1). This invagination and sorting process is called endocytosis, and although several morphologically and structurally distinct endocytic sorting assemblies occur at the surface of most cells [1], perhaps the most recognizable are polyhedral clathrin-coated structures. First identified to be cargo-selective transport carriers during yolk uptake and storage within oocytes of blood-fed mosquitoes [2], clathrin-coated vesicles are now known to support many vital cellular processes, ranging from nutrient uptake, cellular locomotion, and transcriptional regulation and proliferation to complex developmental morphogenetic events. Clathrin-mediated endocytosis also seems important for the efficacy of anti-receptor monoclonal antibody-based tumor therapy [3] and for susceptibility to double-stranded RNA–mediated gene silencing [4].

**Figure 1:**
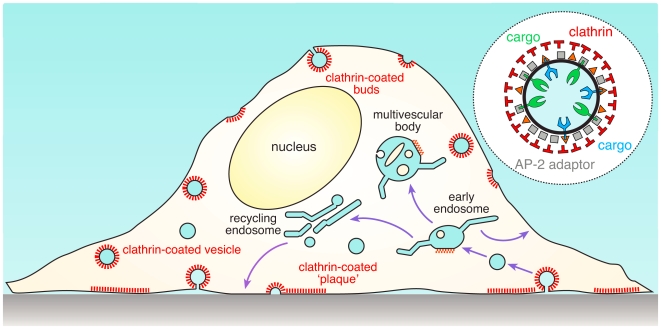
Clathrin-regulated uptake and the endocytic pathway. Schematic illustration of a cultured cell showing surface-positioned clathrin-coated buds, ventrally located large, flat clathrin “plaques,” and the major internal endosomal sorting stations. After clathrin coat uncoating, transport vesicles quickly fuse with the peripheral early endosome compartment, mingling incoming cargo molecules in this initial sorting endosome. Transmembrane cargo can return either directly to the plasma membrane from the early endosome, or be sorted into tubules that are delivered to the juxtanuclear recycling endosome compartment, from which cargo can also be directed back to the cell surface. The bulbous vacuolar portion of the early endosome, containing a flat, bilayered clathrin coat, matures into a multivesicular body for delivery of selected components to lysosomes for degradation. The inset shows the basic composition and organization of a clathrin-coated vesicle, with the three major layers: the inner membrane vesicle with various embedded transmembrane cargo (blue and green), an intermediate layer of adaptors including AP-2 (gray), and the outer clathrin polyhedral lattice (red).

## The Mysteries of Clathrin-Coated Structure Initiation and Function

Clathrin assembles at discrete patches on the plasma membrane through cooperative interactions involving a large set of endocytic proteins [5]. Chief among these are adaptor proteins, which, as the name suggests, link membrane components with the outer layer of the vesicle coat, which is composed of clathrin trimers (Figure 1, inset). While the principal role of clathrin-coated buds is to gather appropriate transmembrane proteins, generically designated “cargo,” for selective delivery to the cell interior, cargo capture is not the driving force for the deposition of coat components at a nascent bud site. AP-2, a major adaptor complex within clathrin-coated structures, has two separate cargo-binding surfaces that are probably both inaccessible when AP-2 first docks onto the plasma membrane [6],[7]. This means that although cargo depends on a sorting signal(s) for its incorporation into clathrin-coated vesicles, recognition of these signals is unlikely to be the event that recruits adaptors and thus allows clathrin coats to form on bare membrane. Instead, the rare and spatially restricted phospholipid phosphatidylinositol 4,5-bisphosphate (PtdIns(4,5)P_2_) seems to play a major role in placing coat protomers on the plasma membrane to begin clathrin assembly [8]–[11]; AP-2 and several other important coat and accessory proteins bind physically to PtdIns(4,5)P_2_ [5]. Perturbing PtdIns(4,5)P_2_ production in cultured cells leads to an almost immediate dissolution of preexisting clathrin-coated structures at the cell surface [8],[10],[11].

**Figure 2:**
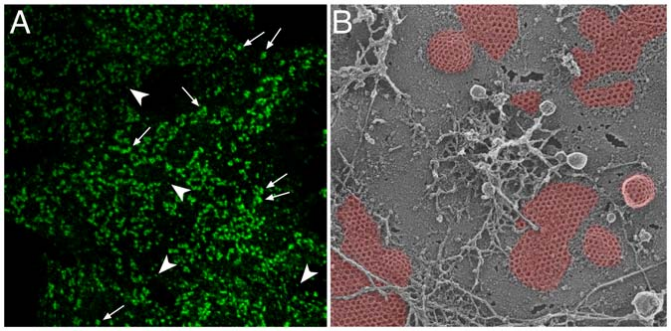
Morphology of clathrin-coated structures at the cell surface. (A) Confocal optical section of a HeLa cell immunolabeled with antibodies to AP-2 to reveal clathrin-coated structures on the adherent plasma membrane. Selected examples of diffraction-limited spots (arrowheads) and large clathrin assemblies (arrows) are shown. (B) Freeze-etch EM image of the adherent surface of a cultured cell, showing both flat and rounded, budding polygonal clathrin structures (pseudocolored in red) on the plasma membrane and the proximity of budding vesicles to the planar sheets.

Because transmission electron microscope (EM) images typically reveal isolated, invaginating coated buds all along the cell surface, and clathrin immunolabeling often shows a profusion of small, separated puncta apparently randomly scattered over the surface membrane (Figure 2), it seems reasonable to suspect that clathrin-coated vesicles might form de novo for each internalization cycle. There is indeed evidence for this from live-cell imaging. In the unicellular yeast *Saccharomyces cerevisiae*, the predictable kinetic behavior of coat components has led to a thorough cataloging of temporally resolved protein entry and exit at single-turnover endocytic sites [12]. In BS-C-1 cells, an African green monkey–derived cell line, clathrin coats at the surface are similarly uniform. The stereotyped behavior of these structures has allowed the definition of coat lifetimes and revealed different types of failure events [13],[14] for these canonical constructions, which are known as clathrin-coated pits. Most importantly, insufficient (or perhaps inappropriate) cargo packaging appears to presage nonproductive collapse of an incipient bud [13],[14]. So, while cargo clearly does not actively recruit coat machinery to the membrane, it plays an important role in driving the process forward to the next step: the budding of vesicles.

Yet, in other cell lines [15]–[19] and isolated primary cells [20], the size distribution of clathrin-coated structures on the cell surface is far less regular than in BS-C-1 cells. For example, in HeLa cells, in addition to transient diffraction-limited objects (<200 nm), large, long-lived and rather sedentary clathrin spots (>500 nm) are observed (Figure 2). Both EM [21],[22] and time-resolved fluorescence imaging techniques, like total internal reflection fluorescence microscopy (TIR-FM) [17],[18],[23],[24], have been used to visualize the bulky clathrin structures on the basal adherent surface, but EM-based visualization of isolated dorsal plasma membrane also shows regions of extensive flat clathrin lattice [25],[26]. Why are there different clathrin assemblies at the plasma membrane and what, if any, is the functional significance of this dichotomy? The variability in position, size, and dynamic behavior of diverse clathrin structures has made global computational analysis of large data sets of time-resolved events very challenging. For one thing, the kinetic inconsistency makes modeling difficult and much depends upon whether, despite morphological and temporal plasticity, the sorting and functional properties of the different patches is basically the same.

## Clathrin-Coated Pits versus Clathrin-Coated Plaques

In this issue of *PLoS Biology*, the Kirchhausen laboratory delves into this issue by utilizing quantitative live-cell imaging of several distinct cell types [27]. Their overarching conclusion is that two mechanistically distinct modes of clathrin assembly and internalization occur at the ventral plasma membrane of different cells. They deduce that rounded buds correspond to de novo–forming, canonical clathrin-coated pits on naked membrane, while clathrin-coated “plaques” are equivalent to the persistent, flat clathrin sheets, and are found only on the basal adherent surface. A pivotal finding is that plaques apparently remain roughly planar throughout, including when an abrupt inward trajectory signifies entry into the cell interior. In curved coated pits, the distribution of AP-2 and epsin, another adaptor, relative to the clathrin coat appears asymmetric, but not in plaques [27]. This again suggests that the underlying plasma membrane is not deformed into a spherical vesicle, typical of most coat-mediated transport events; plaques seemingly maintain a constant arrangement throughout their functional lifetime.

**Figure 3:**
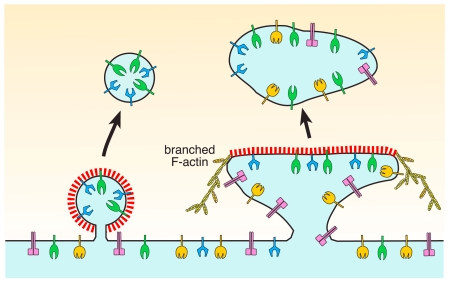
Internalizing clathrin-coated buds and plaques. Schematic depiction of a deeply invaginated clathrin-coated bud and a flat clathrin-coated plaque undergoing endocytic uptake and the resultant uncoated vesicles. Both transmembrane cargo (green and blue receptors) that are selectively sorted into clathrin-coated vesicles and bulk membrane-associated cell surface proteins (orange and magenta) that are perhaps nonselectively incorporated into plaques are shown.

Saffarian et al. provocatively argue that their characterization of general plaque behavior rationalizes a rather discrepant literature, yet the work also raises several fundamental questions. Foremost is whether the plaques they catalog are generally equivalent to the extensive, long-lived, and immobile clathrin patches seen by others. In contrasting ultrastructural studies from other laboratories, the flat patches can be considerably larger [21],[25],[26],[28],[29], and internalization en masse could possibly generate a large puncture in the basal membrane unless much circumferential uncoated membrane is also incorporated into the incoming vesicle, with concomitant reorganization of the underlying surface bilayer (Figure 3). Do plaques invariably enter as intact structures? And, if so, how mechanistically does this occur without compromising the bulk membrane structure? Curiously, despite being plentiful at the adherent surface, thin-section EM images of plaques captured precisely at the instant of internalization are not available. Saffarian et al. assert that the slow relative rate of plaque internalization makes this visualization unlikely. Still, a striking feature of clathrin structures in freeze-etch images of the adherent cell surface is the frequent close proximity of emerging rounded buds to adjacent flat arrays (Figure 2). Corroborating thin-section EM images have been published previously in which buds within, or immediately adjacent to, plaques are plainly seen [21]. One interpretation of this juxtaposed positioning is that flat clathrin arrays could operate as staging scaffolds for spherical bud production. This idea is in accord with observed fluctuations in TIR-FM fluorescence intensity within the persistent lattice population [20], which are compatible with loss of (peripheral) subregions of an extended patch, leaving portions remaining at the plasma membrane [15]. Labeled cargo molecules also have been seen emerging from stationary clathrin patches [20],[26].

Next, we do not understand clearly how, when compared with pits, flat arrays are differentially nucleated and apparently restricted to the adherent surface of the cell. The requirement for a very large excess of hexagonal facets to construct a planar lattice differs obviously from de novo–nucleated buds. Yet, flat lattices must require PtdIns(4,5)P_2_ for assembly, as consumption of this lipid leads to prompt loss of most clathrin structures and adaptors at the ventral surface [10],[11]. Fluorescence recovery after photobleaching (FRAP) experiments indicate that during assembly, clathrin and AP-2 molecules rapidly enter and exit small and large structures alike [16],[30]. That both structures are dynamic at the microscopic level suggests similar overall apparatus and assembly mechanisms. And if cargo is needed to stabilize coats [13],[14], then long-lived plaques must contain and sequester cargo, just as pits do. This is borne out experimentally: fluorescently tagged transferrin (a serum iron transporter that engages the transmembrane transferrin receptor for clathrin-dependent import of iron into the cell interior) labels essentially all surface clathrin structures in HeLa and other cells [17],[19],[20],[30]. The density of transferrin receptor in flat lattice is proportional to receptor concentration [26], and the rate and quantitative nature of transferrin uptake makes it highly unlikely that cargo concentrated within plaques fails to internalize rapidly. Thus, morphologically discernible clathrin structures do appear to cluster the same constitutively internalized cargo.

Another (possibly related) issue is, what prevents the flat clathrin arrays from forming invaginated buds? Perhaps information can be gleaned from comparison with another membrane compartment where flat clathrin arrays also form with no evidence of rounded bud production: on early endosomes maturing into multivesicular bodies (Figure 1). Originally discovered in 1964 [31], we now know that these endosomal clathrin assemblies operate by sequestering cargo, just like clathrin-coated structures at the plasma membrane [32]. However, these so-called bilayered clathrin coats do not contain AP-2 and have an unusual EM morphology that is not seen at plasma membrane plaques. The odd appearance of bilayered clathrin coats could indicate inclusion of structural components that preclude lattice curvature. In canonical, de novo–forming rounded buds, it is thought that the force to sculpt the plasma membrane may come from assembling clathrin, which has inherent curvature when pentagonal facets are incorporated into the lattice [22],[27],[33]; or, instead of inducing curvature itself, clathrin may stabilize curvature resulting from thermal fluctuation–driven membrane rearrangements [34]. Do plasma membrane plaques then, like bilayered clathrin coats on early endosomes, incorporate some additional structural component(s) that blocks clathrin-mediated induction or stabilization of membrane curvature?

An alternative model for what prevents the basally located, flat clathrin arrays from forming invaginated buds is that it is due to a unique role for the actin cytoskeleton in plaque formation, coupled with the strength of adhesion of the basal cell surface to the underlying substrate on which cells are growing. Saffarian et al. find that actin nucleation is unnecessary for the budding of spherical coats, although it is worth noting that at the apical surface of polarized epithelial cells, where clathrin buds are incontestably spherical, actin does regulate vesicle internalization [35] and also drives entry of rounded clathrin-coated structures housing vesicular stomatitis virus [36]. In contrast to what Saffarian et al. observe for bud formation, nucleation of branched actin microfilaments appears to drive plaque movement into the cell [27] (Figure 3). This conclusion is based on the observations that depolymerization of the actin cytoskeleton with the sponge toxin latrunculin A arrests development and internalization of extant plaques and that the branched actin regulators cortactin and Arp2/3 build up at plaques just prior to movement away from the cell cortex [27]. Because latrunculin A application also prevents the formation of new plaques [27], an additional activity of the actin cytoskeleton may be to maintain the planar topology of plaques, perhaps in concert with resistance to deformation caused by tight cell adhesion to the underlying substrate. These observations of actin contributions to plaque formation in particular are intriguing, as the closest mechanistic parallels to the behavior of plaques seem to come from cortical actin patches in *S. cerevisiae*, the sites of clathrin-mediated endocytosis [12]. While absolutely actin-dependent, cortical patches remodel the plasma membrane into tubulovesicular profiles upon internalization [37], quite unlike what is suggested for higher eukaryotic plaques.

Irrespective of mechanism, the current schematic depiction suggests that uptake of a flat clathrin sheet leads to extraneous (noncoated) membrane around the perimeter of the plaque also being internalized (Figure 3). Superficially, this seemingly defies the whole elegant selectivity of coat-mediated sorting. Two important questions arise from this model: how can this membrane excess prevent endocytosis of inappropriate plasma membrane segments, and how is scission regulated at the molecular level?

But perhaps the most important lingering question is what the functional significance of the flat clathrin arrays is, if they have no operative relationship to buds. Two different types of clathrin assemblies could be a physical manifestation of compositionally discrete sorting stations operating in parallel to presort cargo at the cell surface. Data to support the idea of cargo-selective clathrin coats are accumulating [38]–[41], but do not seem to strictly reflect selective partitioning of different membrane-embedded proteins into either pits or plaques. There are, in fact, suggestions that even in BS-C-1 cells, which lack plaques, different types of clathrin sorting structures occur [40]. Doubtless, more work is needed to establish the precise functional interrelationships between different types of clathrin-coated structures, but, unarguably, the new results from Saffarian et al. have given cell biologists much to ponder.

## Abbreviations

EM: transmission electron microscope
PtdIns(4,5)P_2_: phosphatidylinositol 4,5-bisphosphate
TIR-FM: total internal reflection fluorescence microscopy
